# The relationship between cardiac oxidative stress, inflammatory cytokine response, cardiac pump function, and prognosis post-myocardial infarction

**DOI:** 10.1038/s41598-024-59344-5

**Published:** 2024-04-18

**Authors:** Dongpeng Duan, Hongjun Li, Shiyun Chai, Linlin Zhang, Tianfeng Fan, Zhenfeng Hu, Yan Feng

**Affiliations:** 1 Department of Emergency, Affiliated Hospital of Hebei Engineering University, Handan, Hebei China; 2 Publicity and Development Department, Affiliated Hospital of Hebei Engineering University, Handan, Hebei China; 3 Departmentof Renaissance Orthopedics & Otolaryngology Head and Neck Surgery, Affiliated Hospital of Hebei Engineering University, Handan, Hebei China; 4 Department of Infectious Diseases, Affiliated Hospital of Hebei Engineering University, Handan, Hebei China; 5 Department of General Suegery, Affiliated Hospital of Hebei Engineering University, Handan, Hebei China; 6 Department of Critical Care Medicine, Affiliated Hospital of Hebei Engineering University, No.81 Congtai Road, Handan, 056000 Hebei China

**Keywords:** Myocardial infarction, Oxidative stress, Inflammatory cells, Cardiac pump function, Prognosis, Cardiovascular biology, Cardiology, Risk factors

## Abstract

This study delves into the potential connections between cardiac oxidative stress, inflammatory cytokine response, cardiac pump function, and prognosis in individuals following myocardial infarction. A total of 276 patients were categorized into two groups: the control group (n = 130) and the observation group (n = 146), based on the drug intervention strategies. The control group received standard drug treatment, while the observation group received early drug intervention targeting antioxidant and anti-inflammatory treatment in addition to standard treatment. Serum levels of inflammatory factors, including tumor necrosis factor-α (TNF-α), interleukin-1β (IL-1β), and interleukin-9 (IL-6), were assessed using enzyme-linked immuno sorbent assay (ELISA) kits. The Forkhead Box Protein A2 (FOX2) reagent was used to determine the overall oxidation level. Left Ventricular End-Diastolic Diameter (LVEDD), Left Ventricular Ejection Fraction (LVEF), and End-Systolic Diameter (ESD) were measured using Doppler ultrasound. The observation group exhibited significantly reduced serum levels of TNF-α, IL-1β, and IL-6 compared to the control group (*P* < 0.05). Moreover, the observation group exerted lower total oxidation levels, OSI, EDD, and ESD compared to the control group (*P* < 0.05), while the LVEF and TAS levels in the observation group were higher than those in the control group (*P* < 0.05). Remarkably, the observation group experienced a significant reduction in the incidences of reinfarction, heart failure, arrhythmia, and abnormal valve function compared to the control group (P < 0.05). Decreased cardiac pump function and a more unfavorable prognosis were associated with elevated levels of cardiac oxidative stress and inflammatory factors (*P* < 0.05). Timely intervention with appropriate medications have a crucial effect in decreasing inflammatory marker levels, mitigating oxidative pressure, and enhancing cardiac pumping capacity and overall prognosis.

## Introduction

Persistent myocardial ischemia and hypoxia resulting from coronary artery occlusion can lead to the development of myocardial infarction^1^. Despite significant advancements in medical technology and treatments in recent decades, myocardial infarction remains a prominent global cause of mortality. The impact of myocardial infarction on cardiac function and prognosis is substantial^2,3^. Hence, a comprehensive understanding of the pathophysiological alterations following a heart attack and the impact of associated factors on patients’ quality of life and prognosis is of significant importance. Cardiac function and prognosis are profoundly influeced influenced by two fundamental physiological processes that emerge after a myocardial infarction: cardiac oxidative stress and the inflammatory cytokine response^4–6^. The occurrence of a heart attack triggers an ongoing deficiency of oxygen cardiacmuscle tissue due to the blockage in the coronary artery. This, in turn, causes dysfunction in mitochondrial activity and an excessive generation of free radicals^7^. These reactive oxygen species significantly contribute to oxidative stress by causing damage to cellular membranes, proteins, and nucleic acids, ultimately leading to cellular injury and death. Additionally, oxidative stress holds the ability to initiate diverse cellular signaling pathways, exerting a significant influence on the regulation of both physiological and pathological contexts within cardiac cells^7,8^. While the inflammatory reaction serves as a mechanism to maintain tissue homeostasis to a certain extent, an excessive or persistent inflammatory reaction may aggravate heart injury, increase heart stress, and detrimentally impact heart function^9^. Cardiac oxidative stress and the inflammatory cytokine response exhibit a reciprocal relationship. Oxidative stress can stimulate the generation of inflammatory agents, and conversely, inflammatory agents can further escalate oxidative stress^10,11^. This interaction exacerbates the pathophysiological alterations of the heart following a heart attack, significantly influencing cardiac function and prognosis. Consequently, investigating the regulatory mechanism of oxidative stress in the heart and the inflammatory cytokine response post-heart attack, along with their correlation to cardiac function and prognosis, holds substantial clinical and scientific significance. The objective of this research is to investigate the correlation between cardiacoxidative stress, inflammatory cytokine response, cardiac pump function, and prognosis in individuals following a heart attack.

## Materials and methods

### General information

The study included 138 individuals diagnosed with myocardial infarction, admitted to the emergency and cardiothoracic departments of the author’s hospital between February 2021 and November 2022. Participants’ ages ranged from 36 to 70, with a mean age of 62.27 ± 5.46 years. Of the participants, 164 were males and 112 females. Based on prescribed drug intervention protocols, participants were categorized into two groups: the control group (n = 130) and the observation group (n = 146). The control group received standard medication, whereas the observation group underwent early drug intervention targeting anti-oxidation and anti-inflammatory reactions, in addition to the conventional drug treatment provided by the control group. Before being included in the study, all participants were informed about the study procedure and provided their informed consent.

03/08/2023 is the full date of first registration (ChiCTR2300069171).

### Inclusion criteria

Participants were required to fall within the age range of 18–70 years and exhibit ST segment elevation or new left bundle branch block in their electrocardiogram. Additionally, an increase in cardiac biomarkers such as troponin T and the presence of typical symptoms like chest pain or angina pectoris were necessary. The onset of myocardial infarction should not have exceeded 72 h, and individuals needed to express voluntary willingness to participate in the study, accompanied by providing informed consent.

### Exclusion criteria

Exclusion criteria encompassed the presence of severe underlying cardiovascular conditions, such as severe arrhythmia and heart valve diseases. Diagnosis of severe heart failure, concurrent presence of other malignant tumors, and immune system diseases were also grounds for exclusion.

### Ethical information

The research was approved by our hospital’s Ethics Committee, ensuring that all procedures adhered to ethical norms and regulations. Inclusion in the study was contingent upon patients providing written informed consent.

### Drug intervention

Upon admission, every patient received immediate comprehensive care, encompassing procedures such as electrocardiogram (ECG) monitoring, blood pressure measurement, blood oxygen saturation assessment, bed rest, and establishment of venous access, among others. All patients were administered either atorvastatin or rosuvastatin for the plaque stabilization before undergoing emergency PCI. For patients in the observation group, an additional oral dose of aspirin (300 mg) was administered, along with vitamins C and E, to provide anti-inflammatory and antioxidant therapy.

### Analysis of plasma oxidative stress

Upon admission to the hospital, blood samples were obtained and subsequently separated into plasma and serum components. The FOX2 reagent, as previously described by Harma et al. in 2005, was employed to assess the overall plasma peroxide concentration. The preparation of FOX2 reagent involved dissolving ammonium ferrous sulfate in H_2_SO_4_, followed by the addition of butyl hydroxytoluene (BHT) in methanol to the solution. Subsequent addition of xylenol orange led to the formation of the working solution. Plasma was mixed with FOX2 reagent and allowed to incubate at a temperature of 25 ℃. The liquid above the sediment was gathered, and the measurement of light absorption was taken at a wavelength of 560 nm. The Erelmethod (Ozcan, 2004) was applied to determine the total antioxidantstatus (TAS) of the plasma. The antioxidant content of the sample caused the conversion of the colored ABTS radical intoa colorlessform, which was measured as an absorbance at 740 nm. For determination and calibration purposes, a conventional Trolox equivalent known as the standard antioxidant solution was employed. The oxidative stress index (OSI) was defined as the proportion of overall peroxide to total antioxidant status (TAS). The OSI value, measured in arbitrary units, was calculated by multiplying the total peroxide value by 100 and then dividing it by the TASvalue.

### Determination of cytokines

ELISA kit was utilized to measure the levels of serum cytokines TNF-α, IL-1β, and IL-6, following the manufacturer’s instructions.

### Heart pump function evaluation

Siemens PRIME ACUSON ES2000 and Philips EPIQ 7C Doppler ultrasound systems were utilized to measure the left ventricular dimensions, including EDD, LVEF, and ESD.

### The incidence of security events

The incidence of re-infarction, heart failure, arrhythmia, and abnormal valve function were analyzed.

### Statistical analysis

Statistical analysis was conducted using the GraphPad Prism software, based in San Diego, USA. The Shapiro–Wilk test was employed to examine whether the record values in each group followed a normal distribution. Parameter data were analyzed using a one-way ANOVA followed by Tukey test, while non-parametric data were analyzed using a Kruskal–Wallis test followed by Dunn’s multiple comparison test. Correlation among parameters was assesse dusing Pearson and Spearman correlation tests. Significance was considered at p-values equal to or less than 0.05 for all analyses.

## Results

### Patient demographics statistics

Based on the overall patient data, the control group exhibited a male-to-female ratio of 76:54. The average age in this group was 60.21 ± 4.53 years, accompanied by an average BMI of 23.39 ± 1.88 kg/m^2^. Within this group, 36 individuals had hypertension, 14 had diabetes, 40 were smokers, and 32 were alcohol consumers. On the other hand, the observation group included 88 males and 58 females, with an average age of 67.45 ± 5.28 years and an average BMI of 22.57 ± 2.05 kg/m^2^. This group included 40 individuals with hypertension, 12 with diabetes, 46 who smoked, and 36 who consumed alcohol. The general data showed no significant differences between the two groups (*P* > 0.05). (Table 1).

**Table 1:** Statistics of general data of patients ($\overline{{\text{X}}}$ ± s).

Parameter	Control group (n = 130)	Observation group (n = 146)	*T* value*/χ*^2^ value	*P* value
Gender (male: female)	76: 54	88: 58	3.118	0.304
Age (years)	60.21 ± 4.53	67.45 ± 5.28	2.415	0.252
BMI (kg/m^2^)	23.39 ± 1.88	22.57 ± 2.05	3.054	0.355
Hypertension (%)	36 (27.69%)	40 (27.39%)	1.779	0.425
Diabetes (%)	14 (10.76%)	12 (8.21%)	4.526	0.571
Smoking (%)	40 (30.76%)	46 (31.50%)	2.105	0.326
Drinking (%)	32 (24.61%)	36 (24.65%)	1.772	0.105
Serum TNF-α (pg/ml)	33.69 ± 4.56	33.85 ± 5.12	1.120	0.129
Serum IL-1β (pg/ml)	31.02 ± 4.21	31.26 ± 4.63	1.559	0.173
Serum IL-6 (pg/ml)	19.78 ± 3.18	20.36 ± 3.20	1.675	0.152
Total oxides (μmolH_2_O_2_Eq/l)	55.13 ± 3.29	54.32 ± 4.17	1.237	0.138
Total antioxidant status (TAS) (Mmol Total Eq/l)	1.11 ± 0.15	1.05 ± 0.10	1.103	0.118
Serum oxidative stress index (OSI) (AU)	2.98 ± 0.29	2.86 ± 0.25	1.646	0.165
Total cholesterol (TC) (mmol/L)	6.55 ± 2.10	6.61 ± 2.35	1.229	0.126
LDH (U/L)	256.23 ± 23.85	260.51 ± 26.32	1.529	0.165
CK (U/L)	212.99 ± 26.78	219.52 ± 31.26	1.853	0.180
AST (U/L)	49.96 ± 3.86	49.23 ± 5.29	1.237	0.137
The ratio of ST segment elevation to T-wave amplitude(mm)	0.69 ± 0.05	0.72 ± 0.09	1.195	0.120

### Analysis of inflammatory factors in patients

The observation group exhibited markedly decreased serum levels of TNF-α, IL-1β, and IL-6 compared to the control group (*P* < 0.05). (Table 2).

**Table 2:** Serum inflammatory factor level detected by ELISA ($\overline{{\text{X}}}$ ± s).

Groups	TNF-α (pg/ml)	IL-1β (pg/ml)	IL-6 (pg/ml)
Control group (n = 130)	32.41 ± 3.77	28.35 ± 3.41	17.24 ± 2.33
Observation group (n = 146)	23.56 ± 2.49	18.61 ± 1.93	11.53 ± 1.02
*T* value	10.377	9.128	13.054
*P* value	0.001	0.001	0.001

### Analysis of oxidative stress levels

Utilizing the FOX2 reagent, the total oxidation level was determined, and the TAS level was assessed using the Erel method. The observation group exhibited lower total oxidation levels and OSI compared to the control group (*P* < 0.05), whereas the TAS level in the observation group was higher than the control group (*P* < 0.05), as laid out in Table 3.

**Table 3:** Analysis of oxidative stress levels ($\overline{{\text{X}}}$ ± s).

Groups	Total oxide (μmol H_2_O_2_ Eq/l)	TAS (mmol total Eq/l)	OSI(AU)
Control group (n = 130)	51.37 ± 4.28	1.25 ± 0.33	5.83 ± 0.67
Observation group (n = 146)	36.49 ± 3.66	2.47 ± 0.45	2.44 ± 0.31
*T* value	9.102	13.574	11.663
*P* value	0.001	0.001	0.001

### Analysis of patients’ heart pump function

**Figure 1:**
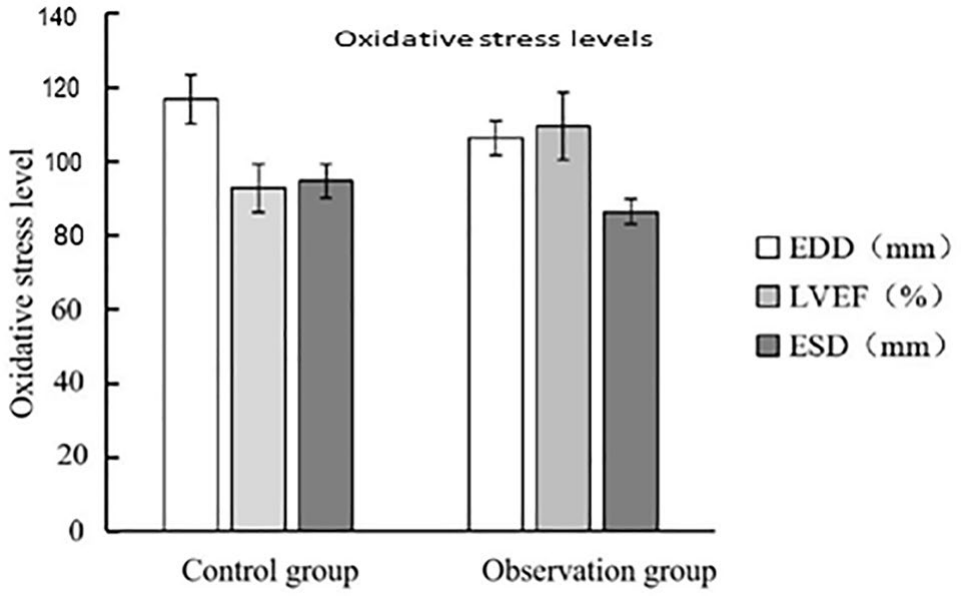
Patient heart pump function.

Doppler ultrasound was utilized to measure the EDD, LVEF, and ESD. The observation group exerted lower EDD and ESD compared to the control group (P < 0.05), while the LVEF in the observation group was elevated in comparison (*P* < 0.05). (Fig. 1, Table 4).

**Table 4:** Analysis of patient heart pump function ($\overline{{\text{X}}}$ ± s).

Groups	EDD (mm)	LVEF (%)	ESD (mm)
Control group (n = 130)	58.38 ± 3.29	46.34 ± 3.29	47.33 ± 2.25
Observation group (n = 146)	53.16 ± 2.37	54.68 ± 4.55	43.18 ± 1.69
*T* value	12.639	11.502	13.007
*P* value	0.001	0.001	0.001

### Analysis of prognosis of patients

**Figure 2:**
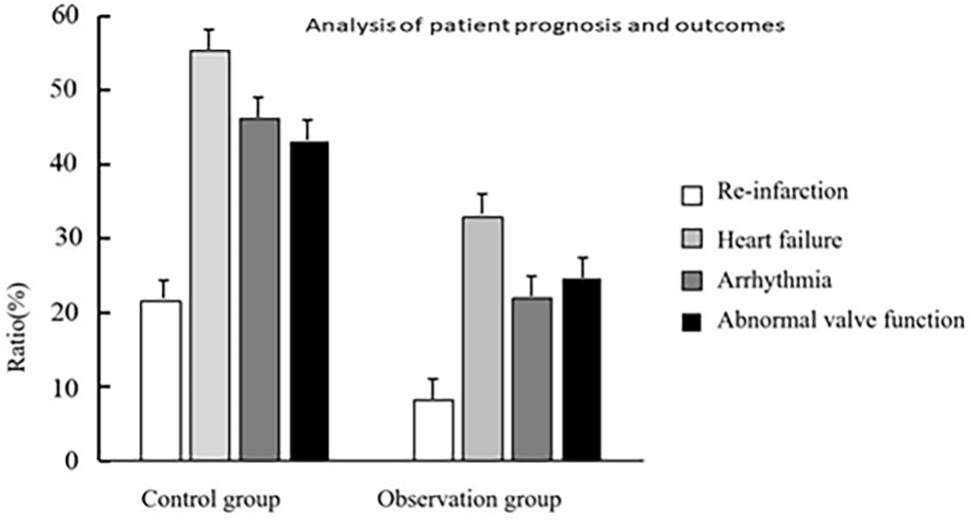
Analysis of prognosis of patients.

In the observation group, occurrences of recurrent heart attacks, cardiac insufficiency, irregular heartbeats, and valve malfunctions were significantly lower compared to the control group (*P* < 0.05). (Fig. 2, Table 5).

**Table 5:** Prognostic analysis of patients ($\overline{{\text{X}}}$ ± s).

Groups	Re-infarction (%)	Heart failure (%)	Arrhythmia (%)	Abnormal valve function (%)
Control group (n = 130)	7 (10.76%)	18 (27.69%)	15 (23.07%)	14 (21.53%)
Observation group (n = 146)	3 (4.11%)	12 (16.43%)	8 (10.95%)	9 (12.32%)
*χ*^2^ value	9.024	11.545	13.619	10.239
*P* value	0.001	0.001	0.001	0.001

### Logistic regression and multiple regression analyses

Through the implantation of logistic regression and multiple regression, the impact of cardiac oxidative stress and inflammatory factors on cardiac pump function and prognosis was investigated. The analysis indicated that these factors played a pivotal role in influencing both cardiac pump function and prognosis following myocardial infarction (*P* < 0.05). Furthermore, a clear correlation was observed between elevated cardiac oxidative stress and inflammatory markers and the decline in cardiac pump function and prognosis (*P* < 0.05). (Table 6).

**Table 6:** Logistic regression and multiple regression analyses ($\overline{{\text{X}}}$ ± s).

Independent variable	β	Standard error	*T* value	*P* value
Cardiac oxidative stress	0.452	0.016	10.503	0.002
Inflammatory factor	0.249	0.003	8.429	0.015
Gender	0.301	0.028	3.118	0.153
Therapeutic regimen	0.116	0.018	4.208	0.337

The meaning of “Oxidative stress in the heart” and “Inflammatory factors” has been added.

## Discussion

Myocardial infarction, a severe cardiovascular disease, commonly triggers myocardial ischemia and necrosis due to the occlusion of coronary arteries. The occurrence of ischemia–reperfusion injury initiates a complex cascade of intricate pathophysiological changes, encompassing the onset of oxidative stress and inflammatory reactions^12,13^. The recovery of cardiac function and prognosis is highly associated with the alterations in cardiac oxidative stress and inflammatory markers following myocardial infarction^14^. When myocardial infarction occurs, the myocardial tissue confronts hypoxia due to coronary artery occlusion, resulting in an insufficient oxygen supply^15,16^. As hypoxia persists, it disrupts the oxidative phosphorylation process within cellular mitochondria, resulting in the excessive production of free oxygen radicals. These oxygen radicals significantly contribute to oxidative stress, posing a threat due to their capacity to damage lipids, proteins, and DNA. In addition, oxidative stress can activate intracellular signaling pathways, possibly triggering inflammatory reaction and apoptosis^17,18^. Following a heart attack, tissue damage and necrosis incite an inflammatory response characterized by the release of inflammatory cells and factors^19,20^. Of note, prominent inflammatory factors including TNF-α, IL-1β, and IL-6 have the potential to activate myocardial cells and attract inflammatory cells, exacerbating myocardial injury and increasing the burden on the heart, with evident impact on cardiac function^21^. The interplay between cardiac oxidative stress and inflammatory factors is intricate. Signal pathways can be activated, potentially inducing the release of inflammatory agents, possibly due to oxidative stress^22^. On the other hand, the presence of inflammatory agents can exacerbate oxidative stress by stimulating the generation of harmful molecules known as free radicals. This interaction worsens the extent of heart damage following a heart attack and impacts the recovery of heart function and overall prognosis^23^. Understanding the dynamic alterations in cardiac oxidative stress and inflammatory markers following a heart attack is crucial for developing targeted treatment strategies. This approach is significant for minimizing cardiac injury, enhancing cardiac function recovery, decreasing the occurrence of cardiac events, and enhancing patients’ prognosis. As a result, addressing cardiac oxidative stress and inflammatory markers has been focused on in the research and management of heart attacks.

This research was undertaken with the objective of elucidating the correlation between oxidative stress in the heart, the inflammatory cytokine response, and their combined influence on cardiac pump function and prognosis following a heart attack. By analyzing 138 patients with myocardial infarction, the study unveiled the impact of cardiac oxidative stress and inflammatory cytokines on cardiac pump function and prognosis after myocardial infarction. The insight garnered from this study greatly contributes to understanding the significance of early drug intervention in treating myocardial infarction.

According to our findings, the observation group underwent prompt medication intervention, leading to a decrease in serum levels of inflammatory markers, including TNF-α, IL-1β, and IL-6. The presence of these inflammatory markers play a crucial role in the pathophysiology following a heart attack, and changes in their concentrations are strongly linked to the extent of cardiac damage, modification in cardiac performance, and overall prognosis. The implementation of early medication intervention aimed at decreasing the levels of inflammatory factors held significant benefits. This approach serves to curtail the inflammatory response, safeguard myocardial tissue, and ultimately improve cardiac function and prognosis. After aheart attack, the substantial inflammatory factors TNF-α, IL-1β, and IL-6 play a significant regulatory role. In fact, activation of these substances can trigger an inflammatory response, resulting in the infiltration of inflammatory cells and subsequent tissue damage. The secretion of inflammatory substances further stimulates signaling pathways, intensifying oxidative stress, inducing lipid peroxidation of cellular membranes, and causing oxidative damage to proteins. These combined effects ultimately exacerbate myocardial damage^24^. Therefore, reducing the magnitude of these inflammatory agents can mitigate the intensity of the inflammatory response following a heart attack, thereby positively influencing the safeguarding of cardiac tissue. By selecting appropriate drug intervention strategies, it becomes feasible to regulate the progression of the inflammatory response, diminish the damage inflicted oncardiac tissue, and facilitate the restoration of normal myocardial function, thereby promoting the process of myocardial repair. Following timely drug intervention, the observation group exhibited significant changes in cardiac oxidative stress. This was characterized by a reduction in the overall oxidation level, a decline in OSI, and an elevation in TAS. These findings underscores the substantial role of timely medication intervention in managing cardiac oxidative stress, thus contributing to its protection against oxidative damage. Cardiac oxidative stress plays a crucial role in the development process after myocardial infarction, and the results of this experiment are consistent with the research of Hao Y et al. The study depicts that due to coronary artery occlusion, myocardial tissue is subjected to hypoxia and reperfusion injury, producing a large amount of free oxygen radicals, which can react with cell membranes, proteins, and nucleic acids, leading to abnormalities in cell structure and function. Oxidative stress can also activate intracellular signaling pathways, leading to inflammatory responses and cell apoptosis^25^. Therefore, reducing cardiac oxidative stress is crucial for protecting the heart from further damage.

Regarding cardiac pump function, subjects within the observation group exhibited reductions in EDD and ESD, along with an elevated LVEF. These findings suggest that early medication intervention could potentially enhance both systolic and diastolic performance of the heart, thereby optimizing blood ejection efficacy from the heart. Furthermore, the observation group manifested significantly lower occurrences of re-infarction, heart failure, arrhythmia, and valve dysfunction compared to the control group, implying that early medication intervention could positively influence patients’ prognosis. Through the utilization of logistic regression and multiple regression analyses, this research further validated the impact of cardiac oxidative stress and inflammatory markers on cardiac pump function and prognosis. Moreover, it discovered a significant correlation between the escalation of cardiac oxidative stress and inflammatory markers and the deterioration of cardiac pump function and prognosis. These findings highlights the pivotal roles played by oxidative stress and the inflammatory response in the pathological progression following myocardial infarction. Our study paves the way for novel treatment strategies in the management of myocardial infarction. Early intervention, directed towards addressing cardiac oxidative stress and inflammatory factors, emerges as a promising avenue for enhancing cardiac function, decreasing the incidence of adverse cardiac events, and ultimately enhancing patients’ prognosis quality. Nevertheless, it is essential to acknowledge that despite significant advancements in research methods and outcomes, certain constraints exist, including a relatively limited sample size and restricted research duration. To further enrich the understanding of myocardial infarction treatment and prognosis, future studies should consider enlarging the sample size, prolonging the research duration, and investigating additional biological mechanisms.

To conclude, our study highlights the crucial importance of cardiac oxidative stress and the inflammatory cytokine response in influencing cardiac pump function and prognosis following a heart attack. Timely drug intervention emerges as a vital factor in decreasing the levels of inflammatory markers, mitigating oxidative stress, enhancing cardiac pumping function, and improving prognosis. This novel perspective on myocardial infarction treatment has the potential to improve patients’ quality of life and mitigate the likelihood of cardiovascular events.

## Data Availability

The data and materials used and/or analysed during the current study are available from the corresponding author on reasonable request.

## Abbreviations

BHT: Butyl hydroxytoluene
ECG: Electrocardiogram
OSI: Oxidative stress index
TAS: Total antioxidantstatus
